# Building a flagellum outside the bacterial cell

**DOI:** 10.1016/j.tim.2014.05.009

**Published:** 2014-10

**Authors:** Lewis D.B. Evans, Colin Hughes, Gillian M. Fraser

**Affiliations:** University of Cambridge, Department of Pathology, Tennis Court Road, Cambridge CB2 1QP, UK

**Keywords:** bacterial flagellum, rotary nanomotor, cell motility, protein export, type III secretion system, chain mechanism

## Abstract

Flagella, the helical propellers that extend from the bacterial surface, are a paradigm for how complex molecular machines can be built outside the living cell. Their assembly requires ordered export of thousands of structural subunits across the cell membrane and this is achieved by a type III export machinery located at the flagellum base, after which subunits transit through a narrow channel at the core of the flagellum to reach the assembly site at the tip of the nascent structure, up to 20 μm from the cell surface. Here we review recent findings that provide new insights into flagellar export and assembly, and a new and unanticipated mechanism for constant rate flagellum growth.

## Highlights

• Cryoelectron tomography reveals details of the intact flagellar export machinery.
• Mechanistic studies reveal discrete stages of the flagellar subunit export pathway.
• Unanticipated chain mechanism for constant rate of flagellum growth.

## The bacterial flagellum: function follows form

**Figure 1:**
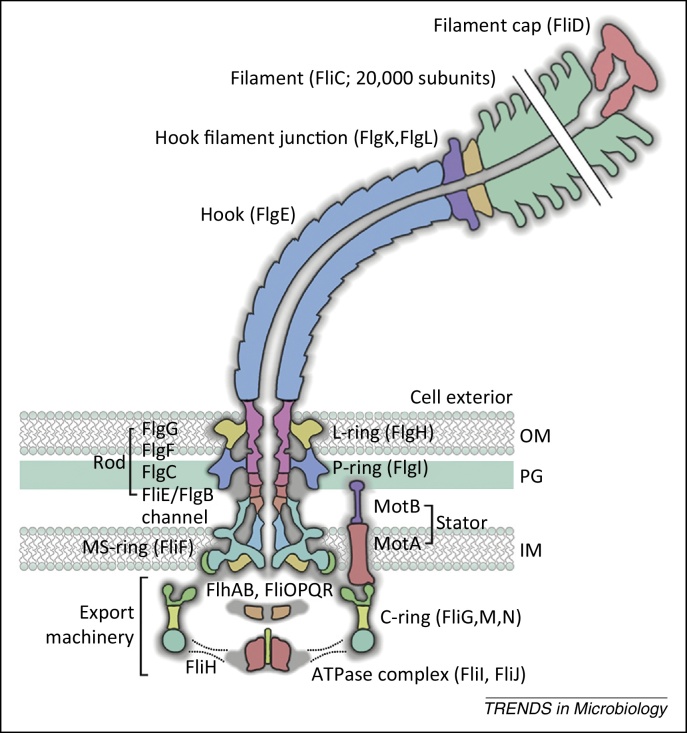
The bacterial flagellum rotary nanomotor. The bacterial flagellum assembles from the inner membrane (IM) to span the PG cell wall and outer membrane (OM), finally extending into the extracellular space. Three contiguous hollow substructures – the rod, hook, and filament – are sequentially assembled. The drive-shaft rod (FliE, FlgB, FlgC, FlgF, and FlgG) is surrounded by a series of rings [lipopolysaccharide (L) ring, FlgH; PG (P) ring, FlgI; membrane–supramembrane (MS) ring, FliF; cytoplasmic (C) ring, FliGMN] and together these elements form the basal body. The C ring interacts with the stator units (MotAB) to drive flagellar rotation. The flexible hook (FlgE) extends from the cell surface with a defined length of ∼55 nm. Hook–filament junction proteins (FlgKL) connect the hook to the flagellar filament (flagellin, FliC). Subunits for the rod, hook, and filament are translocated across the cytoplasmic membrane by a dedicated type III export machinery that comprises an ATPase complex (FliI, FliJ, and FliH; the broken line is the predicted position of FliH), an unfolding cage (FlhAc), and a transmembrane export gate (FlhAB and FliOPQR). On crossing the membrane, subunits then transit through the central channel in the external flagellum to the distal tip, where they crystallise beneath specific cap foldases for the rod (FlgJ), hook (FlgD), and filament (FliD). The positions of individual protein structures are derived from cryoelectron tomography and represent the elements that remain in the mature structure.

The striking relationship of form and function in the flagellum (Figure 1) is the product of evolution over millennia 1, 2, its complex architecture underpinning perfectly its action as a rotary nanomotor that spins freely, both clockwise and counterclockwise, at speeds of up to 100 000 r.p.m. (∼1700 Hz) [3]. This pronounced structure–function link is evident in the three contiguous substructures that comprise the flagellum: the basal body, hook, and helical filament (Figure 1). The basal body spans the bacterial cell envelope and comprises a ‘drive-shaft’ rod and a series of rings. Inside the cell, the basal body broadens into a bell-like structure called the cytoplasmic (C) ring or switch. This is the rotor part of the motor and is also the input point for signals that control the direction of flagellar rotation and, consequently, cell movement [4]. Studded around the basal body are the stator units of the motor. The rotor and stator components work together to harness the electrochemical energy of proton or sodium ion-motive forces, powering flagellum rotation [5]. Motor composition is dynamic, with individual stator units recruited to the basal body in response to changes in the ion-motive force and the mechanical load on the flagellum 6, 7, 8, 9.

The basal body C ring connects to the membrane–supramembrane (MS) ring, which has a central channel and socket-like region in which the rod is anchored [10]. In Gram-negative bacteria, two further rings, designated L (lipopolysaccharide) and P [peptidoglycan (PG)], act as bushings for the rotating structure and allow the rod to penetrate the cell envelope [11]. Attached to the basal body rod and extending from the cell surface is a short, curved hook that functions as a flexible universal joint [12]. Contiguous with this is the long, rigid helical filament ‘propeller’, constructed from thousands of flagellin subunits to extend 15–20 μm from the cell surface [13]. Motor rotation is transmitted through the rod and hook to the filament and, in a mechanism similar to an Archimedean screw, the rotating helical filament engages with the fluid medium to generate linear thrust that pushes the cell forwards [14].

A remarkable feature of the flagellum is that it is essentially self-assembling. At the base of each flagellum, housed in a cavity bounded by the MS and C rings, a dedicated type III export machinery (Figure 1) unfolds newly synthesised structural subunits and translocates them across the cell membrane into a narrow 2 nm channel that spans the full length of the nascent structure [15]. This first stage of export is energised by ATP hydrolysis and the proton motive force (PMF) 16, 17. Once in the channel, the unfolded subunits must transit the entire length of the external flagellum to reach its tip, where they crystallise beneath cap structures 18, 19, 20, 21. In this way, the proximal rod is built first, followed by assembly of the hook and then the filament. Remarkably, the rate of flagellum growth appears to be independent of flagellum length [22] and, until recently, the mystery has been how this constant-rate growth of the external flagellum is energised.

In this review we discuss recent work that has provided new insights into flagellum export and assembly. Structural details of the intact flagellar export machinery have been revealed and mechanistic analyses have given a clearer picture of the discrete stages of the subunit export pathway and set out a new and unexpected explanation of how flagella could grow at a constant rate.

## Surveying the scene: imaging the export machinery *in situ*

Recent developments in cryoelectron tomography have provided the first detailed images of flagella in their cellular contexts 23, 24, 25. In particular, these studies have revealed the locations of export machinery components [23] (Figure 1). At the flagellum base, the transmembrane domains of six export components (FlhA, FlhB, FliO, FliP, FliQ, and FliR) form an export gate dome that bulges from the centre of the MS ring into the cytoplasm [23]. The export gate aligns with the central channel in the MS ring, which transitions from a closed to an open state when subunit export is initiated [25]. Positioned 6–10 nm beneath the export gate dome, a homononameric ring of the FlhA cytoplasmic domain (FlhA_C_) forms a toroidal platform that is connected to the membrane export gate by thin linker domains 23, 26. It was initially thought that this platform also contained the cytoplasmic domain of the FlhB export gate component (FlhB_C_), but its loss did not disrupt the toroid [26] suggesting a different location for FlhB_C_, possibly in the export gate dome. Together, the membrane export gate, linker, and platform form the boundaries of an ‘export cage’ situated immediately beneath the membrane [26]. A spherical density 10 nm beneath the export cage comprises the homohexameric FliI export ATPase 23, 27. FliI associates with two other proteins, FliJ and FliH, and this ATPase complex has homology to the F_1_ domain of the F_0_F_1_ ATP synthase [28]: FliI is homologous to the F_1_ α and β subunits, and FliJ and FliH are homologues of the F_1_ central stalk γ subunit and peripheral stator δ subunit, respectively 29, 30. The observed distance between the ATPase complex and the export cage suggest that when the export machinery is inactive these structures do not interact [27]. However, when the export machinery is active, it is proposed that the FliJ central stalk spans the gap between the ATPase and the export cage to interact with FlhA 26, 31, 32. Similarly, the elongated FliH protein is thought to anchor the ATPase complex to the surrounding C ring 26, 33.

## Unravel to travel: subunit targeting to and unfolding by the export machinery

**Figure 2:**
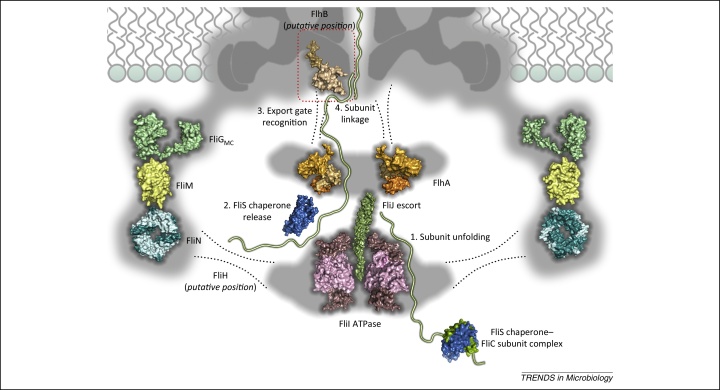
Sequential interactions of flagellar subunits with the membrane export machinery. The rotary ATPase complex (FliI hexamer, 2DPY [67]; FliJ escort, 3AJW [28]) is associated with the C ring (FliG_MC_, 1LKV [68]; FliM, 2HP7 [69]; FliN tetramer, 1YAB [70]) via FliH (proposed position indicated by broken lines). The chaperone–subunit complex (FliS–FliC, 1ORY [71]) docks initially at the ATPase complex before entering the export cage, comprising a nonameric ring of FlhA (FlhA_C_, 3A5I [72]), and then passing to the export gate component FlhB_C_ (3B0Z [45]). Subunits are then translocated across the cell membrane into the export channel.

To enter the export pathway (Figure 2), flagellar subunits synthesised in the cytoplasm must be targeted to the membrane export machinery and this is achieved, in part, by export signal sequences in the subunit N terminus 34, 35, 36. Subunits initially engage the export ATPase complex, where they are thought to be unfolded, at least partly, before being presented to the export cage and membrane gate 35, 37. For subunits of the flagellar filament, hook–filament junction, and filament cap (but not the rod or hook), specific chaperones appear to be critical for targeting to the membrane export machinery and, specifically, initial docking at the ATPase 15, 37, 38, 39, 40. Chaperoned subunits then pass to the FlhA_C_ component of the export cage, where again the chaperones are essential in establishing high-affinity interactions 32, 41, 42. The chaperone–subunit complexes bind specifically to a conserved hydrophobic dimple at the interface of the cytoplasmic D1 and D2 domains of FlhA_C_. Remarkably, the binding affinities reflect the order in which the subunits are assembled into the nascent flagellum, with chaperoned hook–filament junction subunits (FlgN–FlgK) binding with a slightly higher affinity than chaperoned filament cap subunits (FliT–FliD), which in turn bind to FlhA_C_ with a 14-fold higher affinity than chaperoned flagellin (FliS–FliC) [42]. This differential binding could increase the efficiency of filament assembly by promoting the export of subunits for the junction and cap structures, which must assemble before flagellin can polymerise. This mechanism is augmented by FliJ, which further enhances the binding of junction and cap subunits to the export cage by recruiting their cognate chaperones to FlhA_C_ 32, 43. By analogy with the F_1_ γ central stalk, FliJ interactions with the export cage are proposed to be dynamic, with rotation of the FliJ stalk possibly driving conformational changes in FlhA_C_ that facilitate both chaperone release and further unfolding of subunits to aid subsequent membrane translocation [26].

## Border crossing: subunit recognition at the export gate and membrane translocation

While studies of chaperoned subunits have revealed mechanistic principles underlying the early stages of export involving the ATPase complex and the export cage, work on the unchaperoned subunits of the rod and hook has uncovered details of later steps that require the FlhB export gate component. FlhB has two domains connected by a flexible linker, the integral membrane FlhB_TM_ domain, which comprises four transmembrane α helices, and the cytoplasmic FlhB_C_ domain, which, unusually, undergoes autocleavage at a conserved NPTH motif [44]. Once cleaved, the resulting FlhB polypeptides remain associated 45, 46. Cleavage is critical for full export function, as mutation of the cleavage site locks FlhB in a conformation that permits export of only rod and hook subunits, abolishing export of filament subunits [47]. This structural flexibility appears to be an important feature of FlhB_C_ as other mutations that similarly limit flexibility also reduce subunit export 48, 49.

Recruitment of subunits to the export gate requires a surface-exposed hydrophobic pocket on the cleaved FlhB_C_ C-terminal polypeptide (FlhB_CC_) [36]. This pocket could provide a binding site for a conserved hydrophobic gate-recognition motif (with sequence Fxxxϕ, where ϕ is any hydrophobic residue) in the N-terminal region of rod and hook subunits that is essential for FlhB binding and export [36]. Binding of subunits to FlhB_C_ *in vitro* appears to be relatively weak, with dissociation constants in the micromolar range, reflecting the transient nature of subunit interactions at the export gate [36].

**Figure 3:**
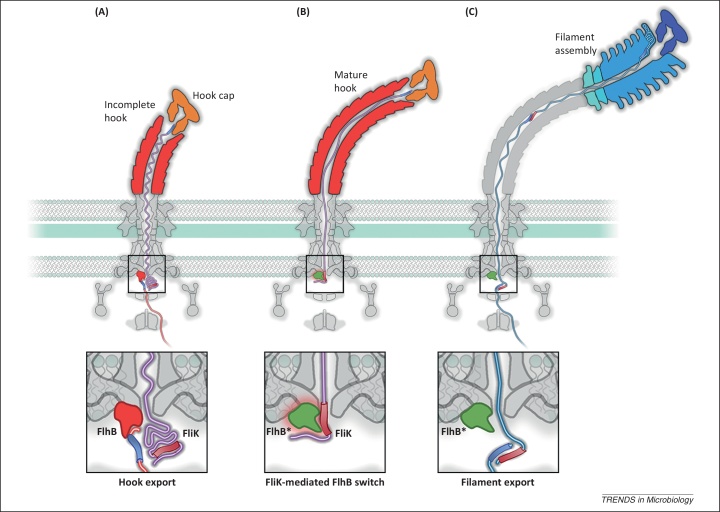
Model for hook-length control and the export-specificity switch mediated by the molecular ruler FliK. **(A)** During assembly of the flagellar hook (red), export of hook subunits is interrupted intermittently by the export of the molecular ruler FliK (purple). The N terminus of FliK interacts with the hook cap (FlgD, orange) at the tip of the nascent hook and, before the hook reaches its mature length of ∼55 nm, the C-terminal region of the transiting FliK does not interact with the FlhB export gate, which is instead occupied by incoming hook subunits. **(B)** When the hook reaches its mature length, a gate-binding region (red cylinder) in the C terminus of the transiting FliK becomes exposed and remains in the cytoplasm for a sufficient length of time to form a productive interaction with the FlhB export gate, causing a conformational change (FlhB*). The precise mechanisms underlying this export-specificity switch are unknown. **(C)** The specificity switch permits the export, and subsequent assembly, of filament subunits (blue).

In addition to binding structural subunits of the rod and hook, the FlhB_C_ export gate is bound by FliK, an unusual export substrate that senses the length of the flagellar hook and, when the hook has reached its mature length, switches the specificity of the export machinery to recognise subunits for the flagellar filament (Figure 3) [50]. FliK contains a canonical N-terminal gate-recognition motif that binds the surface-exposed hydrophobic pocket on FlhB_C_ [36], but also contains a highly conserved C-terminal region with an acidic loop (residues 294–300, LHPEELG) that is proposed to bind to a basic patch adjacent to the FlhB_C_ autocleavage site and thus control export-specificity switching [51]. The precise mechanism of specificity switching is not fully understood, although several models have been proposed (and are discussed in recent commentaries 52, 53). The available data are, perhaps, best explained by FliK acting as an infrequent molecular ruler that is exported intermittently during rod and hook assembly 54, 55, 56. In this model, as hook length increases so does the probability that FliK will form a productive interaction with FlhB to flip the export-specificity switch and promote export of filament subunits (Figure 3).

Once docked at the export gate, how do subunits then move across the membrane into the central channel in the growing flagellum? Energy for membrane translocation seems to be provided, in part, by the PMF 16, 17, and the contributions of its two components – the electrical potential difference (ΔΨ) and the proton gradient (ΔpH) – appear to be separable 16, 17, 57. Translocation energised by ΔpH is postulated to be an intrinsic property of the export gate, with a conserved aspartate (Asp208) in FlhA suggested to be involved, perhaps indirectly, in proton transport [58]. Harnessing the energy of ΔΨ, however, requires the ATPase complex 57, 58. Specifically, interactions between the FliJ ATPase central stalk with the FlhA export cage are needed for the export gate to function as an efficient ΔΨ-driven protein translocator [57]. Although the mechanism of PMF-powered membrane translocation remains unclear, it is proposed that ΔΨ and ΔpH act together to energise the export gate in its suggested function as a proton–protein antiporter [57].

## Journey to biological outer space: subunit transit through the external flagellum

Once subunits have crossed the membrane into the central channel of the external flagellum, how do they reach the assembly site at the tip of the structure, which can be several cell lengths away? Until recently, it was thought that subunits moved through the central channel in the external flagellum by passive diffusion, with the rate of flagellum growth slowing exponentially as the structure lengthened 59, 60, 61. However, a landmark study by Berg and colleagues [22] showed that, as the flagellum lengthens outside the cell, the rate of filament growth does not change, with one subunit crystallising into the structure about every 2 s. This constant rate of flagellum growth precludes diffusion of unfolded subunits through the channel because if one new subunit enters the channel every 2 s and the rate of subunit diffusion is much slower than this (it is estimated that it takes about 10 s for an unfolded subunit to diffuse 1 μm), subunits would gradually accumulate in the channel [36]. In this ‘crowded regime’, multiple interactions between subunits would generate an increasing resistance force that would decrease the subunit transit rate and the channel would eventually become clogged [62]. However, what if, as proposed in a recent theoretical model [63], subunits remain partially folded in the channel: could diffusion support constant-rate flagellum growth? In this model, transiting subunits adopt an unprecedented extended α-helical fold, a notion counter to the observation that subunits in the central channel of the related virulence needles are unfolded [64]. Moreover, the model does not take into account the resistance forces generated by the observed interactions between subunits in the channel [36]. These resistance forces would increase as the flagellum lengthened, slowing the rate of growth [62]. This indicates that a constant rate of subunit transit through the channel in the external flagellum cannot occur by passive diffusion but must be energised. But, where does the energy come from?

**Figure 4:**
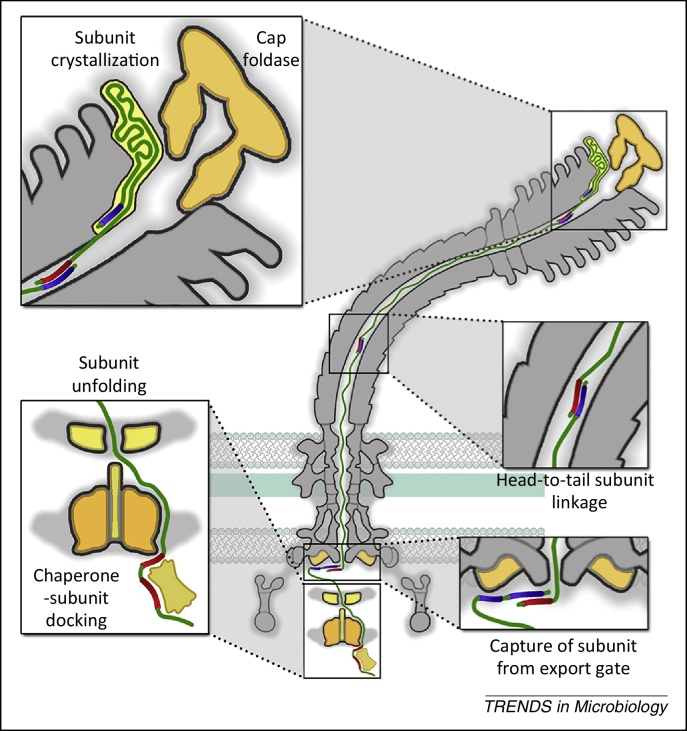
A chain mechanism delivers subunits to the assembly tip of the external flagellum. Subunits are unfolded by the export machinery before recognition by the FlhB_C_ export gate component. The N terminus (blue) of the subunit docked at the export gate is captured by the free C terminus (red) of an exiting subunit in the flagellar channel, linking subunits head to tail in a chain. Linked unfolded subunits in the channel transit to the flagellum tip, where they crystallise sequentially beneath the cap foldase. As subunits fold into the flagellum tip, the chain becomes stretched, increasing the entropic pulling force at the cell-proximal end of the chain until a threshold force is reached and a new subunit is pulled from the export machinery into the channel. This process repeats, delivering subunits to the flagellum tip at a constant rate.

It is now apparent that the energy for transit is intrinsic to the unfolded subunits themselves as they move from the export machinery across the membrane and into the channel (Figure 4). Transit is achieved by linking of the subunit docked at the FlhB export gate to the free C terminus of the preceding subunit that has already partially crossed the membrane into the central channel in the external flagellum [36]. The juxtaposed N- and C-terminal helices of successive subunits are predicted to form a parallel coiled coil, to which each subunit contributes 14–32 residues. Newly linked subunits are then pulled from the gate into the flagellar channel by the thermal motion of the unfolded subunit chain, which is anchored at its other end to the tip of the nascent structure (Figure 4). Repeated folding of subunits into the tip produces directional subunit transit and causes the chain to shorten and stretch, thus exerting an increasing pulling force on the next subunit at the gate, eventually pulling it into the channel. The vacated export gate is then available to bind the next incoming subunit, which again links into the growing chain. In this way, successive rounds of subunit linking at the membrane export machinery are coupled to subunit crystallisation at the tip to allow continuous subunit transit and constant-rate flagellum growth [36]. This chain mechanism for flagellum growth imposes strict requirements on the forces underlying each stage of subunit passage; specifically, that subunit anchoring at the tip must be stronger than the links between subunits in the chain, which in turn must be stronger than subunit binding to the membrane export gate, as confirmed by thermodynamic analysis [36].

Subunit crystallisation at the tip of the flagellum is clearly central to the generation of the pulling force that drives subunit transit through the channel. Most structural subunits crystallise beneath cap structures that associate with the distal tip of the nascent flagellum during assembly. Three distinct caps participate in assembly: the FliD filament cap; the FlgD hook cap; and the FlgJ rod cap, which has muramidase activity that allows the rod to penetrate the PG cell wall 18, 19, 20. The rod and hook caps are temporary structures that are discarded on completion of their cognate substructures. Only the filament cap, comprising a pentamer of FliD, remains in the mature flagellum [65]. The FliD oligomer has a distinctive pentagonal plate structure from which extend five leg domains that bind the flagellum tip 18, 65. Underneath the cap plate, and surrounded by the cap ‘legs’, there is a cavity that is apparently large enough to accommodate a single flagellar subunit. This cavity is thought to act as a folding chamber, similar to an Anfinsen cage [66]. A symmetry mismatch between the filament, which has helical 11-fold symmetry, and the pentameric cap means that four of the leg domains each make a structurally distinct contact with the flagellum tip, with one leg unable to bind the tip. It is in this transient gap, formed between two of the cap leg domains, that subunits crystallise into the structure. Once a subunit has inserted, the cap leg domains go through a series of asymmetric conformational changes and the cap then rotates to open the next gap for crystallisation of a newly arriving subunit. The thermal energy of subunit incorporation is thought to power cap rotation [18]. The binding of crystallised subunits to the flagellum is very strong and cannot be broken spontaneously by thermal motion. Thus, subunit crystallisation provides a strong anchor point for the subunit chain and drives directional subunit transit through the channel [36].

## Concluding remarks and future directions

The studies described above have unveiled new facets of export machinery function and an unprecedented ‘intrinsic’ mechanism allowing flagellum growth beyond the cell surface. They have also opened new avenues of investigation into mechanistic aspects of flagellum biogenesis that are currently unresolved (Box 1). Questions surround the ordered assembly of structural subunits, as the mechanisms that control the sequence of subunit incorporation into the rod, hook, and filament are unknown – could order be established at the membrane export machinery or imposed by subunits’ affinities for each other as they link into the transiting chain? How is the more evident export-specificity switch between export of rod/hook and filament subunits achieved? Further work is also required for a deeper understanding of how the export ATPase and PMF work together to power subunit translocation across the cell membrane.

Box 1 **Outstanding questions**

• How is the sequential assembly of rod, hook, and filament subunits controlled?
• What is the mechanistic basis of the export-specificity switch controlled by FlhB and FliK?
• How do the integral membrane export components facilitate subunit translocation across the membrane?
• How do the PMF and the flagellar export ATPase energise subunit membrane translocation?
• Do transiting subunit chains contain a mix of subunits?
• What happens when the transiting chain of subunits breaks?
• Does the chain mechanism operate in related type III secretion systems for needle assembly?

There are several unanswered questions about the chain mechanism, the first being what happens if the subunit chain breaks? This could cause flagellum growth to pause as the most cell-distal subunit in the broken chain diffuses to the flagellum tip, where it would fold to re-establish the chain mechanism. A second question concerns the composition of the subunit chain and, specifically, whether the chain contains a mix of subunits linked head to tail (e.g., flagellin and cap subunits), each of which is required at different stages of assembly. On reaching the tip, surplus subunits will fold as they exit the channel but will not crystallise into the structure; this is observed as accumulation of, for example, discarded monomeric cap subunits in the extracellular environment. A further question is whether the chain mechanism operates in the assembly of the type III secretion needles used to deliver virulence effectors into eukaryotic host cells. Needles are evolutionarily related to flagella [2] and, as in flagella assembly, unfolded structural subunits are translocated across the bacterial membrane before they transit through a narrow central channel and incorporate at the needle tip. Could the helical termini of needle subunits link as parallel coiled coils to form a subunit chain? Little is known about the growth dynamics of needles and further work is required to determine whether the chain mechanism operates during needle assembly. Future studies addressing these and other outstanding questions will undoubtedly lead to a deeper understanding of how large macromolecular machines can be assembled outside living cells and may throw new light on other assembly problems.
